# Merging Porphyrins with Gold Nanorods: Self Assembly Construct to High Fluorescent Polyelectrolyte Microcapsules

**DOI:** 10.3390/nano12050872

**Published:** 2022-03-05

**Authors:** Vanda Vaz Serra, Sofia G. Serra, Mariana C. S. Vallejo, Pedro M. R. Paulo, Nuno M. M. Moura, David Botequim, Maria Graça P. M. S. Neves, Sílvia M. B. Costa

**Affiliations:** 1 Centro de Química Estrutural, Instituto Superior Técnico, Universidade de Lisboa, Av. Rovisco Pais, 1049-001 Lisboa, Portugal; sofiagserra@sapo.pt (S.G.S.); davidbotequim@gmail.com (D.B.); sbcosta@tecnico.ulisboa.pt (S.M.B.C.); 2 LAQV-REQUIMTE, Department of Chemistry, University of Aveiro, 3810-193 Aveiro, Portugal; mariana.vallejo@ua.pt (M.C.S.V.); nmoura@ua.pt (N.M.M.M.); gneves@ua.pt (M.G.P.M.S.N.)

**Keywords:** porphyrins, metal nanoparticles, polyelectrolyte microcapsules, fluorescence imaging, plasmonic antennas

## Abstract

Dual probe porphyrin-gold nanorod polyelectrolyte microcapsules were developed to explore the enhancing effects of a plasmonic interface of self-assembled gold nanoparticles in the fluorescence emission from porphyrins loaded into the capsules’ core. An analysis of fluorescence lifetime imaging microscopy (FLIM) data reports a notable 10^5^–10^6^-fold increase in the maximum detected photon rates from diffraction-limited spots and an overall six-fold increase in fluorescence as averaged over the whole microcapsule area. Large emission enhancements were correlated with decreases in fluorescence lifetimes. The microcapsule’s design proved effective in achieving high fluorescent hybrids and may shed light on new possibilities for advanced materials imaging applications.

## 1. Introduction

In recent years, polyelectrolyte microcapsules (PECs) have received a great deal of attention due to their possible use as drug delivery systems, biosensors, and micro-reactors [1,2]. Using the adequate polymers and number of assembled layers, fine control over a PEC’s structure can be achieved, allowing for devices with high biocompatibility and mechanical/chemical stability [1,3,4,5], which can load hydrophobic and hydrophilic molecules and promote their controlled and targeted release [6,7,8].

The integration of dyes, such as porphyrins, within these platforms enables the production of optically addressable devices and expands PECs’ use towards fields involving light. Porphyrins are important light-absorbing molecules that are largely recognized as photosensitizers for cancer photodynamic therapy and diagnosis due to their intrinsic ability to generate cytotoxic oxygen species and to emit in the red or near infrared when exposed to visible or UV light [9]. Nevertheless, most porphyrins are weak emitters, have solubility issues, and tend to aggregate in water [10,11], thus hampering most cases for their application in biomedicine. Porphyrinoids’ functionalization with hydrophilic units [12,13,14,15] is an elegant synthetic strategy used to overcome porphyrins’ low solubility. Nanotechnological development, is also encouraging the use of different nanoparticles such as gold nanoparticles (AuNP), as porphyrinoid carriers to improve imaging and photodynamic capabilities [16,17,18,19].

Bédard et al. were the first to incorporate porphyrins in the PEC’s shell and to study the use of ^1^O_2_ formed upon light irradiation to promote dye release [20]. Other examples include the studies of the doping procedure impact and shell nature on the optical and electronic properties of porphyrins [21,22] and as templates for the construction of supramolecular porphyrin assemblies [11,23]. Laser-responsive AuNP photothermal effect has been used to remotely study a triggered PEC’s fusion [24], polymer exchange phenomena [25], macromolecules’ release upon irradiation with short laser pulses in the near-infrared region [26], and photothermal therapy [27].

Concerning porphyrinoid-gold assemblies, most studies thus far refer to a gold core coated by one layer of the dye which is directly linked to the nanoparticle surface via thiol chemistry, electrostatic interactions, or adsorbed onto silica, polyelectrolytes shell/films, or DNA oligonucleotides [28,29,30,31,32,33,34,35,36].

Plasmonic engineering of fluorescence enhancement of weak emitters is a matter of active research. Despite the variety of plasmonic materials employed for fluorescence enhancement, gold nanoparticles are by far the most used due to their chemical stability and the well-established procedures for their synthesis and functionalization. Gold nanoparticles can act as antennas for visible light and confine the electromagnetic field in a small volume near its surface, increasing the absorption of light from a nearby molecule. The excitation rate is proportional to the intensity of light and, thus, is accelerated by the local electric field enhancement near the particle. The particle’s proximity also opens additional nonradiative decay channels that have quenching effects on the dye’s emissions. One process that plays a crucial role is the resonant energy transfer from the dye to the particles’ plasmon modes, which is then dissipated as heat. Therefore, the above-mentioned emission enhancements are strongly dependent on the local field enhancement induced by the nanoparticle on the relative position and distance between organic molecules and metal nanoparticles and their spectral overlap. In particular, gold nanorods have the advantage that their longitudinal plasmon resonance can be tuned across the NIR region by changing their aspect ratio. Fluorescence microscopy is often used to evaluate emission enhancement effects from single dye molecules. In these examples, the dye freely diffuses in a liquid medium so that it can explore the optimal location of plasmonic hot-spots [35,37], or the dye is entrapped in a polymer film and many individual plasmonic nanostructures are probed to find the strongest enhancement effects via an optimal dye-particle configuration [38,39]. An earlier work reports on the successful deposition of a phthalocyanine within a lipid vesicle adsorbed on the surface of a modified microcapsule with dispersed gold nanospheres [21], but the metal enhanced fluorescence effect was not observed probably due to high heterogeneity and poor spectral overlap.

**Figure 1:**
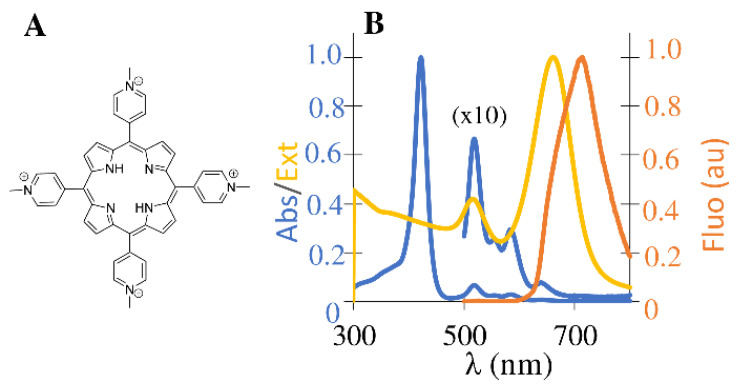
(**A**) Molecular structure of tetracationic porphyrin TMPyP. (**B**) Normalized absorption (blue) and emission (orange) spectra of TMPyP in water; normalized extinction spectra (yellow) of gold nanorods in water.

**Scheme 1:**
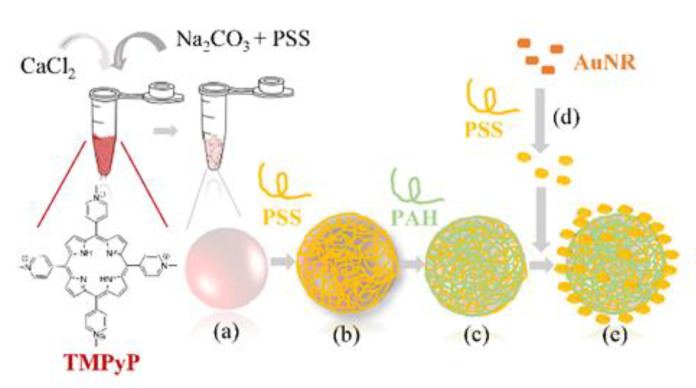
Preparation of porphyrin-nanogold PEC’s hybrid via co-precipitation and layer-by-layer mixed approaches: (**a**) porphyrin embedded CaCO_3_ microparticle CaCO_3_-TMPyP; (**b**,**c**) wrapping of oppositely charged polyelectrolytes leading to porphyrin embedded PECs (CaCO_3_-TMPyP)-PSS and (CaCO_3_-TMPyP)-PSS-PAH, respectively; (**d**) coverage of gold nanorods, AuNR, with negative polyelectrolyte PSS; (**e**) Porphyrin-nanogold PEC’s hybrid (CaCO_3_-TMPyP)-PSS-PAH-(PSS-AuNR).

Bearing in mind the development of highly fluorescent micro-nanomaterials for bioimaging applications herein, we have expanded the concept of fluorescence enhancement of organic dyes from a different perspective by exploiting the multi-compartmentalization features of smart polyelectrolyte capsules. In the pursuit of this goal, we have designed a porphyrin-nanogold interface and characterized its enhanced emission properties. One of the major reasons to focus on PECs as all-in-one devices is that their synthesis is herein based exclusively on layer-by-layer methodologies to avoid organic coupling while bringing together easily scalable processes in water. The weak emitter 5,10,15,20-tetrakis(*N*-methylpyridinium-4-yl)porphyrin (TMPyP) was arbitrarily distributed within the PECs core and the microcapsule outer-shell was used as a template for building a plasmonic interface of randomly self-assembled gold nanorods (AuNR) with multiple nanogap hotspots (Scheme 1). The observed spectral overlap between AuNRs’ longitudinal mode and TMPyPs’ absorption (Q bands) and emission spectra, enables the direct excitation of both dye and plasmon oscillation (Section 3.1, Figure 1). Therefore, we would expect a resonant plasmonic enhancement of porphyrin’s molecular emission. Still, TMPyP and AuNR may exhibit small differences in absorption and emission due to encapsulation. The optical properties of these hybrids were investigated using conventional spectroscopies. FLIM was used to probe unequivocal evidence of remarkably high porphyrin enhanced emission.

## 2. Materials and Methods

### 2.1. Materials

Poly(sodium 4-styrenesulfonate) [PSS (MW ≈ 75,000, 18 wt% in water)] and poly(allylamine hydrochloride) [PAH (MW ≈ 15,000)], Figure S1, were obtained from Sigma-Aldrich (Steinheim, Germany). Trifluoroacetic acid (TFA) and sodium hydroxide (NaOH) used to control the pH were also purchased from Sigma-Aldrich. CaCl_2_ (purity ≥ 99.5%)] was obtained from Fisher Chemical (Waltham, MA, USA) and Na_2_CO_3_ was obtained from J. T. Baker (Philadelphia, PA, USA). TMPyP tetraiodide salt was purchased from Sigma-Aldrich. Gold nanorods (CTAB stabilized, 25 × 71 nm, product number A12-25-650-CTAB-DIH-25 lot RPD236AD) were purchased from Nanopartz Inc., Ltd. (Loveland, CO, USA). All the reagents were used as received. Polyelectrolyte solutions (3 mg/mL of PSS and 12 mg/mL of PAH, 0.5 M NaCl) were prepared in bi-distilled water, and the pH was adjusted to 6.5 for the PSS/PAH used in the PEC’s preparation. PSS solution (1.7 mg/mL) was used for nanorod functionalization. Glass microscope slides (0.13–0.17 mm thickness) were obtained from Normax, Marinha Grande, Portugal.

### 2.2. Porphyrin-Nanogold PEC’s Hybrid Preparation

Porphyrin-nanogold PEC hybrids were prepared according to the following steps:

(i) Porphyrin embedded CaCO_3_ template preparation and polyelectrolytes wrapping (Scheme 1a–c). Equal volumes of 100 μL of saturated solutions of Na_2_CO_3_ (0.33 M with PSS 18 wt%) and CaCl_2_ (0.33 M) were added under intense stirring to 50 μL of an aqueous solution of TMPyP (260 μM). After 30 s, the mixture was allowed to rest for 15 min. Porphyrin doped CaCO_3_ microparticles were obtained after supernatant removal, followed by three washing/centrifugation cycles (3000 rpm, 2 min) to remove any non-encapsulated dye. Similar procedures in the absence of porphyrin were used to prepare nonfunctionalized CaCO_3_ PECs. CaCO_3_ microparticles (with or without TMPyP) were then dispersed in an aqueous solution of PSS (1 mL, 3 mg/mL, 0.5 M NaCl). After stirring for 15 min, the resulting particles were centrifuged (6000 rpm, 10 min), and the supernatant was removed. After three washing/centrifugation cycles to remove residual PSS, the obtained microparticles were dispersed in an aqueous solution of PAH (1 mL, 12 mg/mL, 0.5 M NaCl). After the usual washing/centrifugation procedures, PECs were obtained free from residual PSS and stored in bi-distilled water.
(ii) Gold nanorods adsorption onto porphyrin embedded PECs (Scheme 1d–e): PSS wrapped gold nanorods: AuNR solution (1 mL, OD = 1) were centrifuged (6000 rpm, 15 min). After supernatant removal, PSS polyelectrolyte (1 mL, 1.7 mg/mL, 1 mM NaCl) was added. The mixture was vigorously stirred and after 1 h it was centrifuged and washed with bi-distilled water (3 cycles, 6000 rpm, 15 min) to remove the excess of PSS. These PSS-wrapped gold nanorods were then resuspended in 500 µL of bi-distilled water and added to previously prepared porphyrin embedded polyelectrolyte microcapsules. After the usual washing/centrifugation steps, porphyrin-nanogold PECs hybrids were stored in bi-distilled water.

### 2.3. Characterization

UV-Vis absorption measurements were performed in a PerkinElmer Lambda 35 spectrophotometer (PerkinElmer Inc., Ltd., Waltham, MA, USA). Corrected fluorescence measurements were recorded with a Fluorolog-3 spectrophotometer (Horiba Jobin Yvon, Tokyo, Japan). The cuvettes used for spectrophotometry were quartz cuvettes from Hellma (optical path length 10 mm, 700 μL volume). The effective zeta potential values were measured in a Zetasizer Nano ZS Doppler electrophoretic light scattering analyzer from Malvern Instruments Inc., Ltd. (Malvern, UK). The pH was measured at 24.0 °C with a Crison microph 2002 (Crison, Barcelona, Spain). The samples were centrifuged using a Spectrafuge 24D Digital Microcentrifuge (Labnet, Orange, CA, USA), with a centrifuging radius of 8.23 cm. Samples for electron microscopy were prepared by dispensing one or two drops of PECs dispersed in water on carbon-coated copper grids and were allowed to dry at room temperature overnight. Scanning transmission electron microscopy (STEM) measurements were performed on a TEM/STEM Hitachi microscope model HD2700 (Schaumburg, IL, USA) operating at 200 kV, equipped with EDS and HAADF detector. Scanning electron microscopy measurements were performed with Analytical FEG-SEM equipment JEOL 7001F (JEOL Inc., Ltd., Tokyo, Japan) with an Oxford light elements EDS detector and an EBSD detector. FLIM measurements were performed with a time-resolved confocal microscope (MicroTime 200, PicoQuant GmbH, Berlin, Germany). Cover-slips were carefully cleaned prior to use. Cover glasses were first immersed in an RBS solution (5%) for 2 h followed by rinsing with ultrapure water. After drying, cover slips were exposed to UV/Ozone (equipment PSD-UV3 from Novascan) for 2 h. Briefly, excitation at 639 nm was carried out by pulsed diode laser heads at a repetition rate of 20 MHz. Band-pass filter 695AF55 was used for the red diode laser. The fluorescence lifetimes were detected with a single-photon-counting avalanche diode (SPAD) (PerkinElmer Inc., Ltd., Waltham, MA, USA) whose signal is processed by a TimeHarp 200 TC-SPC PCboard (PicoQuant GmbH, Berlin, Germany) working in time-tagged time-resolved (TTTR) operation mode. The emission intensity time traces were collected from random points, chosen within PECs during a time interval of 180 s, using an excitation power of ca 0.44 kW/cm^2^ to avoid porphyrin’s photobleaching.

### 2.4. Discrete Dipole Approximation Simulations

The method of discrete dipole approximation (DDA) was used [40,41] to model plasmonic hot spots between closely packed AuNRs and the enhancement effect on porphyrin’s emission. A full detailed description of the calculation procedures can be found elsewhere [35,42]. Firstly, the near field maps were calculated for a set of particle configurations comprising two, four, and seven nanorods, in order to identify the possible plasmon hot spots. The shortest gap distance between nanorods was set at 3 nm to account for the polymer coating in the experiment. The model nanorods are spherically capped cylinders with a diameter of 25 nm and length of 61 nm that were discretized as an array of cubic elements with a side of 0.5 nm. The dielectric function of gold was used as reported by Johnson and Christy [43]. Then, the porphyrin was modeled as a point-like dipole emitting at specific wavelengths, each representing a spectral component within the dye’s emission spectrum [37]. The plasmon-enhanced radiative and non-radiative decay rates for the emitter in the plasmon hot spots were calculated using the theoretical formalism described by D’Agostino et al. [42].

## 3. Results and Discussion

### 3.1. Preparation and Characterization of Porphyrin-Nanogold Polyelectrolyte Microcapsules Hybrids

TMPyP is a tetracationic porphyrin known for its high singlet oxygen quantum yield (Φ_∆_ = 0.7 in phosphate-buffered solution), a noteworthy affinity for single and double-strand DNA polyanion [44]. It is highly selective, targeting cancer cells over non-tumorous cells in a wide variety of established cancer models [45,46]. TMPyP has a molar absorption coefficient (ε) value of around 10^3^ M^−1^ cm^−1^ at the excitation wavelength of 639 nm and a low fluorescence quantum yield of 5% due to the mixing of the S1 state with a charge transfer state (CT) involving an electron transfer pathway between the N atoms of the pyrrolic units and the *meso* pyridinium substituents which has been well covered in other studies [47] Due to four *N*-methylpyridinium *meso*-substituents, this porphyrin is water soluble and able to self-assemble through electrostatic interactions with negatively charged polyelectrolytes, the primary constituents of the polyelectrolyte shell. Figure 1 shows the optical spectra of individual TMPyP and AuNRs that were obtained in water before encapsulation in PECs.

The absorption spectrum of TMPyP displays a Soret maximum at 422 nm (from S_0_ → S_2_ transition) and four satellite bands in the 500–700 nm region (from S_0_ → S_1_ transition). In general, free base water soluble monomeric porphyrins present a characteristic emission spectrum with two vibrational bands, Q(0,0) and Q(0,1), around 650 nm and 720 nm, respectively. The unresolved emission spectra of the porphyrin TMPyP is explained by the above-mentioned CT state [47]. AuNRs have two plasmon modes; the transversal surface plasmon resonance (SPR) mode at 518 nm, excited by the light polarized along the rod’s transverse direction, and the longitudinal SPR mode at 658 nm, which is associated with electron oscillations across the rod’s long axis [48] that gives rise to large nearfield enhancements. The observed spectral overlap between AuNRs’ longitudinal mode and TMPyPs’ absorption (Q bands) and emission spectra, enables the direct excitation of both dye and plasmon oscillation. Therefore, we would expect a resonant plasmonic enhancement of porphyrin’s molecular emission. Still, TMPyP and AuNR may exhibit small differences in absorption and emission due to encapsulation.

The preparation and characterization of the porphyrin-nanogold PEC hybrids will be discussed in detail in the next sections, following the summarized procedure in Scheme 1 (Section 2.2).

The typical design of PECs doped with low molecular dyes usually starts with the dyes’ adsorption on the polyelectrolyte shell by taking advantage of their ionic character. In this work, an alternative co-precipitation approach was used to promote porphyrin encapsulation within the CaCO_3_ core matrix during microparticle growth (spectroscopic characterization details can be found in Table S1). Afterward, the full coverage of TMPyP-CaCO_3_ microparticles with a bilayer of PSS-PAH polyelectrolytes (Figure S2) took place, using well-known layer-by-layer methods [11,22] to prevent TMPyP release and provide a fair nanometric control over the distance between porphyrin and AuNRs. Commercially available AuNRs are positively charged due to the presence of a bilayer of CTAB (ζ = +30 mV) [49,50], and therefore are not suitable for PAH adsorption without further functionalization.

In our case, the modification of the entire nanorod surface is preferable to its tip functionalization (where plasmonic hot spots are located) [51,52,53] since a higher number of attaching points will increase the contact area, strengthening AuNR adsorption onto a (CaCO_3_-TMPyP)-PSS-PAH surface. Due to the polymeric nature of PECs, AuNR overcoating with PSS [49,50], which introduces sulfonate groups at their surface which allows for their adsorption to positively charged -NH_3_^+^ groups in the PECs last layer, seems to us a natural choice. Furthermore, polyelectrolyte coverage significantly reduces CTAB cytotoxicity and increases AuNRs’ stability in water [52,54,55].

Our UV-Vis data documented a 3 nm red shift (from 658 to 661 nm, Figure S3) in the longitudinal plasmon mode after PSS adsorption, which is consistent with the UV-Vis changes reported in reference [49]. Zeta potential measurements also support this, as a charge reversal is observed from +29.5 ± 5 mV to −29.9 ± 2 mV.

**Figure 2:**
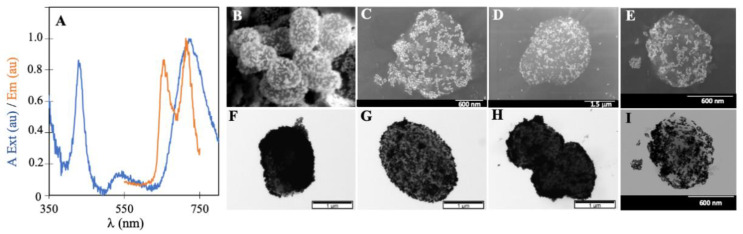
Porphyrin-nanogold (CaCO_3_-TMPyP)-PSS-PAH-(PSS-AuNR) PEC’s hybrid. (**A**) Absorption (blue) and emission (orange) spectra in water. (**B**–**E**) SEM and (**F**–**I**) TEM images of typical PEC’s hybrid, showing gold nanorods bright (**B**–**E**) and dark (**F**–**I**) nanometric structures randomly distributed on the surface of porphyrin embedded polyelectrolyte microcapsules (micrometric round structures; dark (**B**–**E**) and bright (**F**–**I**)). Images (**E**) and (**I**) are, respectively, SEM and TEM images of the same microcapsule.

The as-prepared AuNR-PSS was added to a known amount of (CaCO_3_-TMPyP)-PSS-PAH (Scheme 1, Section 2.2), and a change in the PEC’s color from light red to dark blue was observed. The UV-Vis spectrum of the porphyrin-nanogold PEC hybrids (Figure 2A) in water presents the typical signatures of TMPyP and AuNR entities, confirming their successful encapsulation: a TMPyP Soret band around 432 nm, a broad band between 450–650 nm attributed to a superposition of TMPyP Q bands and the transverse plasmon mode, and a NIR band at 717 nm that originates from the longitudinal plasmon mode.

It is important to note that neither the width nor the wavelength maxima of TMPyP in PEC hybrids are changed by the adsorption of polyelectrolytes or by the proximity of the AuNRs, which means that the porphyrin remains intact during the experiment. However, significant changes are observed in the AuNR’s longitudinal mode peak wavelength with a red shift from 661 to 717 nm (56 nm), due to the combined effects of changes in the dielectric constants of AuNRs adsorbed and coupling of plasmon resonances from neighboring AuNRs which are closely adsorbed in the same microcapsule [56].

Calculation of the amount of AuNR-PSS adsorbed based on UV-Vis spectra of the supernatant cannot be readily applied in this instance, due to aggregation of unbound AuNR-PSS. However, taking as reference an approximate value for the molar absorption coefficients (ε~10^5^ M^−1^ cm^−1^ for TMPyP (420 nm) [57]; ε~10^9^ M^−1^ cm^−1^ for the AuNRs LSPR (605–845 nm)) [58], the relative ratio of AuNR to TMPyP in (CaCO_3_-TMPyP)-PSS-PAH-(PSS-AuNR) was roughly estimated to be 1:8000. TEM and SEM images of the as-prepared porphyrin-nanogold hybrids (Figure 2B–I and Figures S2E,F and S4), reveal a dense packing of AuNRs within the micrometric PEC’s surface.

### 3.2. Plasmonic Fluorescence Enhancement in Polyelectrolyte Microcapsules Hybrid

**Figure 3:**
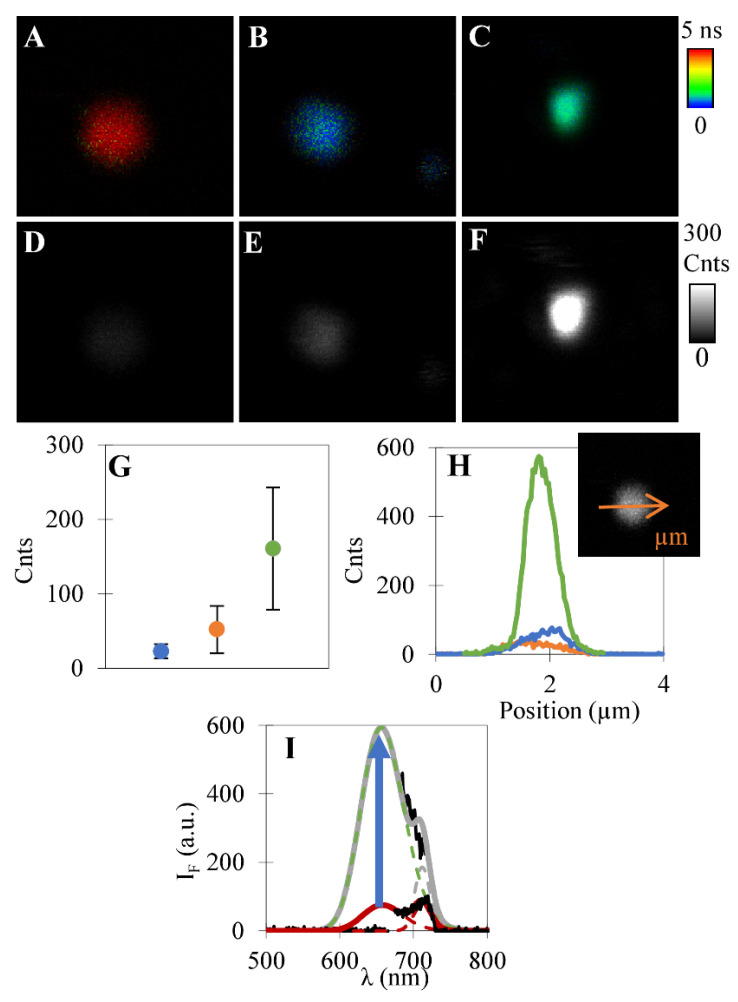
Examples of typical FLIM and intensity images (5 × 5 µm) of: (**A**,**D**) Porphyrin functionalized PECs (CaCO_3_-TMPyP)-PSS-PAH; (**B**,**E**) gold functionalized PECs (CaCO_3_)-PSS-PAH-AuNR; (**C**,**F**) porphyrin-nanogold PEC’s hybrid (CaCO_3_-TMPyP)-PSS-PAH-(PSS-AuNR). (**G**) Average counts calculated for (CaCO_3_-TMPyP)-PSS-PAH (orange), (CaCO_3_)-PSS-PAH-(PSS-AuNR) (blue), and (CaCO_3_-TMPyP)-PSS-PAH-(PSS-AuNR) (green). (**H**) Intensity profiles of the orange arrow for images (**A**) (orange), (**B**) (blue), and (**C**) (green). (**I**) Photoluminescence spectra of TMPyP in PECs (CaCO_3_-TMPyP)-PSS-PAH (non-enhanced emission-red) and in porphyrin-nanogold PEC’s hybrid (CaCO_3_-TMPyP)-PSS-PAH-(PSS-AuNR) (enhanced emission-grey) from images (**A**,**C**).

The antenna effect of the plasmonic interface of randomly assembled AuNRs on the porphyrins’ emission was evaluated by FLIM. Images of more than 30 individual (CaCO_3_-TMPyP)-PSS-PAH-(PSS-AuNR) particles (5 × 5 µm) were collected using laser excitation of λ_exc_ = 639 nm (0.44 kW/cm^2^), which closely matches the longitudinal plasmon resonance of AuNRs, and the absorption from TMPyP Q bands (Figure 3C,F and Figure S5). The same type of image analysis was performed on two control samples prepared accordingly but containing only TMPyP or only AuNR, organized as (CaCO_3_-TMPyP)-PSS-PAH (Figure 3A,D) and CaCO_3_-PSS-PAH-(PSS-AuNR) (Figure 3B,E).

The average fluorescence enhancement for porphyrin-nanogold PEC’s hybrid (160.9 counts) was experimentally determined after subtraction of the background signal of unenhanced porphyrin emission (average number of counts of control (CaCO_3_-TMPyP)-PSS-PAH 23.1 counts)—Figure 3G.

According to these data, we estimate an average enhancement factor of six times more fluorescence from TMPyP in porphyrin-nanogold PEC’s hybrids due to their plasmonic outer layer of AuNRs. This is a remarkable result since it translates the overall picture of compact or sparse regions of AuNRs within a single capsule, and it also accounts for local heterogeneity between different PECs resulting from small differences in the surface distribution of AuNRs. The photoluminescence spectra obtained for porphyrin embedded PECs with and without plasmonic layer (green and red curves, respectively, Figure 3I), also reflect a significant increase in the number of photon counts/ms measured in the hybrid.

**Figure 4:**
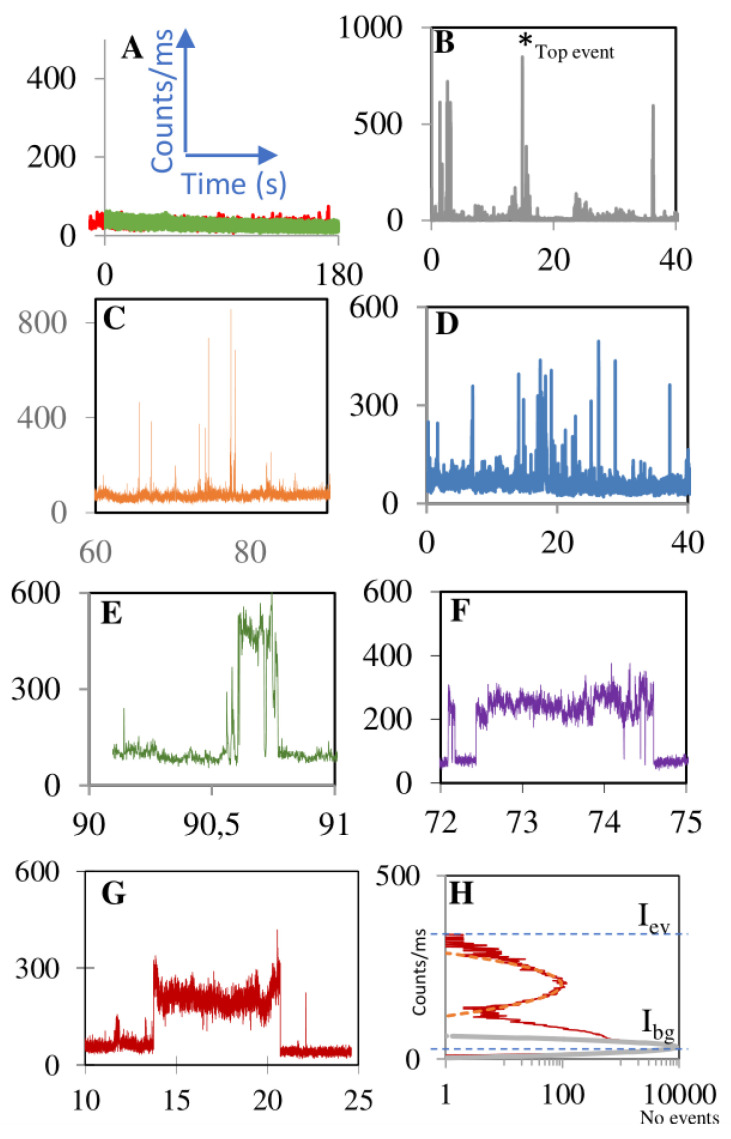
Examples of typical intensity time traces found for: (**A**) Controls; PECs containing only porphyrin (green) or gold nanorods (red) and for (**B**–**H**) porphyrin-nanogold hybrids. In (**B**), one example of the events of higher intensity from fluorescence enhanced porphyrins is signed with (*).

We have also used FLIM to probe maximum local fluorescence enhancement factors and compare our results with other works from the literature. Several fluorescence time traces were recorded in randomly chosen diffraction-limited spots of individual porphyrin-nanogold PECs and controls (CaCO_3_-TMPyP)-PSS-PAH and CaCO_3_-PSS-PAH-(PSS-AuNR). Figure 4 shows various sections of different emission behavior extracted from typical fluorescence time traces acquired in our hybrids, that display strong and discrete fluorescence emission events of much higher intensities than the trace baseline. Sometimes these events are several consecutive bursts (B to D) while in other cases they are prolonged emission events (E to G). Note that strong emission is absent in the fluorescence time traces measured for controls. Therefore, the events observed in time traces B to G (Figure 4) are attributed to fluorescence enhancement of porphyrin emission.

While our measurements are not strictly limited to single-molecule fluorescence, because many porphyrins are present within the confocal volume, the discrete characteristic of these intense emission events suggests that these may arise from a single porphyrin molecule passing through interparticle gap hot-spots and, consequently, experiencing a strong enhancement effect on its emission. The stochastic nature of these events is reflected in the number, intensity, and duration of fluorescence bursts that vary from time-trace to time-trace. This result was expected because gold nanorods are randomly dispersed on the PEC’s surface. Below we further discuss this issue based on DDA simulations. Additionally, different orientations and gap separations between neighbor gold nanorods may give rise to plasmonic hot spots with significant differences in the plasmon near-field enhancements that affect the strength of the antenna effect. Furthermore, the polyelectrolyte bilayer that separates the porphyrin (core) from the gold nanorods offers an ionic barrier that is likely to slow down porphyrin diffusion in the vicinity of gold nanorods, allowing TMPyP to probe local electromagnetic fields that translate into different fluorescence enhancements.

The top enhancement factor was calculated from the intensity of the strongest emission event (I_ev_) corrected from the background signal I_bg_ (intrinsic photoluminescence of individual porphyrins and AuNRs in the same capsule) and normalized to the intensity of one emitter in the absence of the antenna (I_0_), according to Equation (1):

F/F_0_ = (I_ev_ − I_bg_)/I_0_ (1)

For porphyrin-nanogold PEC’s hybrid, the top fluorescence bursts obtained from 30 independent fluorescence time traces were found to range from 345,000 to 954,000 counts/s with an average I_bg_ of 50,000 counts/s. Considering that, in the same experimental conditions, the non-enhanced emission intensity (I_0_) of a single porphyrin is 0.8 counts/s [35], one can estimate that the top events measured range from 10^5^ to 10^6^ more photons detected during the events of enhanced emission than those obtained from TMPyP alone. Such large enhancement factors are in the same range as those reported earlier for the same porphyrin in gold nanodimer antennas made of gold nano-spheres linked through a DNA spacer [35]. Moreover, the maximum enhancement found here compares favorably with those reported in studies on 10^3^-fold emission increase for crystal violet in the vicinity of a single AuNR [37] or a 10^4^-fold increase in the emission of the same molecule in end-to-end assemblies of AuNRs [59].

To confirm the singularity of the (CaCO_3_-TMPyP)-PSS-PAH-(AuNR-PSS) design, microcapsules containing both porphyrin and AuNR now co-precipitated in the same CaCO_3_ core and covered by a bi-layer of polyelectrolytes (CaCO_3_-TMPyP-AuNR)-PSS-PAH were also prepared. No evidence of plasmonic fluorescence enhancement was found, probably due to a low amount of AuNRs adsorbed (Figures S6 and S7).

### 3.3. DDA Simulations

**Figure 5:**
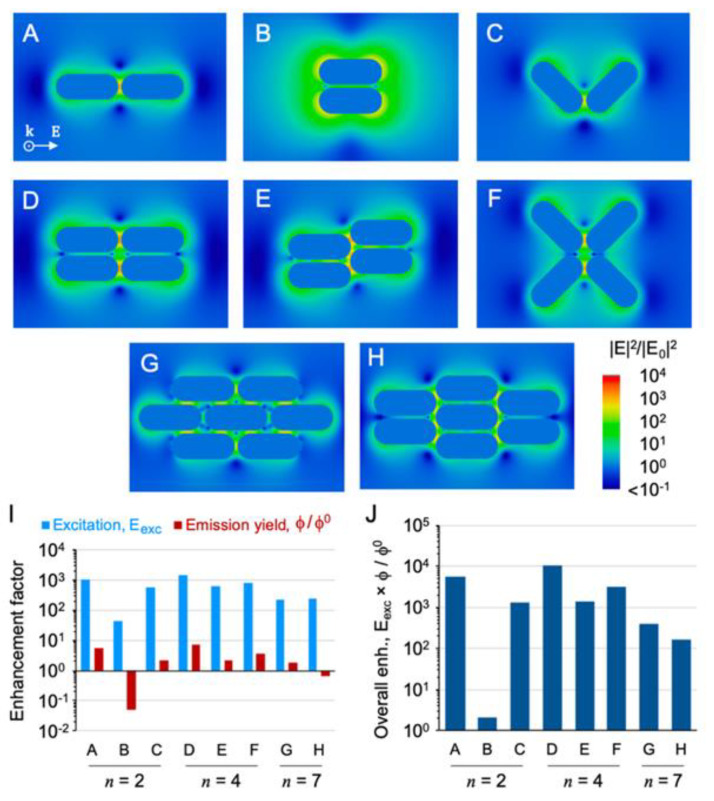
Model simulations of plasmonic hot spots between AuNRs for particle configurations of (**A**–**C**) two, (**D**–**F**) four, and (**G**,**H**) seven nanorods. (**I**) Calculated enhancement factors for excitation rate, E_exc_, and relative emission quantum yield, Φ/Φ^0^. (**J**) Overall emission enhancement calculated for a porphyrin-like emitter in selected hot spots of simulated particle configuration (see text for further details).

We have performed DDA simulations of a set of particle configurations idealized as models of coupled AuNRs in the plasmonic outer layer of PECs (Figure 5A–H) to confirm that emission enhancements of several orders of magnitude are attainable for a porphyrin dye interacting with nanogap hot spots.

For each particle configuration, these maps were used to identify hot spot regions, and, therein, a position was defined for a point-like dipole emitter that simulates the emission of TMPyP (Figures S8–S10). Another set of simulations were performed, now in the spectral range of the dye’s emission, which estimates the relative emission quantum yield, Φ/Φ^0^, i.e., the ratio between the modified quantum yields due to the particle’s antenna effect and the dye’s intrinsic one. In most configurations, the selected hot spot positions allowed for the enhancement of Φ/Φ^0^ below 10-fold, which may seem modest, however, this factor is intrinsically limited to 20-fold (Figure 5I). The excitation rate enhancement, E_exc_, with estimated values that can reach up to 1000-fold is then the prevailing factor for the overall emission enhancement, E_exc_ × Φ/Φ^0^. The overall enhancement factors obtained from these model simulations reach a 10^4^-fold emission increase (Figure 5J) for comparable conditions to those used in the experiments. The latter has shown that larger emission enhancements are experimentally observed. A similar difference between simulation and experiment has been observed before, for single porphyrin’s emission enhanced by nanogap antennas [35], which deserves a more detailed investigation in future works.

## 4. Conclusions

In summary, herein we show that a compartmentalized structure of polyelectrolyte microcapsules can be used as efficient platforms to create spectrally engineered microsystems for plasmonic fluorescence enhancement of weak emitters. Particularly, the assembly of AuNRs strongly enhances porphyrin emission, resulting in an overall PECs fluorescence enhancement of six times and on fluorescence enhancements from discrete emission events, which are 10^5^ to 10^6^-fold. To the best of our knowledge, this is the first example of PECs being used as a depot system to maintain and supply a defined level of weak emitters in the vicinity of gold nanoantennas. This result is an important step towards designing and improving devices targeting a wide range of fluorescence imaging applications.

## Data Availability

Not applicable.

## Supplementary Materials

The following supporting information can be downloaded at: https://www.mdpi.com/article/10.3390/nano12050872/s1, Table S1: Absorption (λ_max_^A^) and emission (λ_max_^F^) maxima wavelengths of TMPyP in water or PSS solution and TMPyP doped CaCO_3_ microparticles (CaCO_3_-TMPyP), Figure S1: Molecular structures of PSS and PAH, Figure S2: SEM images of non-functionalized PECs, one probe polyelectrolyte microcapsules and Porphyrin-nanogold PEC’s hybrid, Figure S3: Normalized extinction spectra of AuNR and respective PSS wrapped AuNR, Figure S4: Representative TEM images of TMPyP-nanogold PEC’s hybrid, Figure S5: Examples of typical FLIM images measured for porphyrin-nanogold PEC’s hybrid, Figure S6: Representative TEM and SEM images of alternative PEC’s hybrid obtained by co-precipitation of both TMPyP and gold nanorods, Figure S7: Representative FLIM images obtained for TMPyP doped PECs, Figure S8: Model simulations of plasmonic hot-spots between AuNRs, Figure S9: Near-field enhancement maps calculated from model simulations, Figure S10: Calculated spectra of particle configurations shown in Figure S8 or Figure S9.
