# Supplementary material for: Merging Porphyrins with Gold Nanorods: Self Assembly Construct to High Fluorescent Polyelectrolyte Microcapsules
Source: Nanomaterials (Basel). 2022 Mar 5;12(5):872. doi: 10.3390/nano12050872 (PMC8912806; doi:10.3390/nano12050872)
Supplement: Supplementary file 1 [file nanomaterials-12-00872-s001.zip › nanomaterials-1610243-supplementary.pdf]

nanomaterials

Supplementary Materials

Merging Porphyrins with Gold Nanorods: Self Assembly Construct to High Fluorescent Polyelectrolyte Microcapsules

Vanda Vaz Serra [1,*], Sofia G. Serra [1], Mariana C. S. Vallejo [2], Pedro M. R. Paulo [1,*], Nuno M. M. Moura [2], David Botequim [1], M. Graça P. M. S. Neves [2] and Sílvia M. B. Costa [1]

[1] Centro de Química Estrutural, Institute of Molecular Sciences, Instituto Superior Técnico, Universidade de Lisboa, Av. Rovisco Pais, 1049-001 Lisboa, Portugal; sofiagserra@sapo.pt (S.G.S.); davidbotequim@gmail.com (D.B.); sbcosta@tecnico.ulisboa.pt (S.M.B.C.)
[2] LAQV-REQUIMTE, Department of Chemistry, University of Aveiro, 3810-193 Aveiro, Portugal; mariana.vallejo@ua.pt (M.C.S.V.); nmoura@ua.pt (N.M.M.M.); gneves@ua.pt (M.G.P.M.S.N.)
* Correspondence: vanda.serra@tecnico.ulisboa.pt (V.V.S.); pedro.m.paulo@tecnico.ulisboa.pt (P.M.R.P.)

Table S1. Absorption (λmaxA) and emission (λmaxF) maxima wavelengths of TMPyP in water (TMPyP/Water) or PSS solution (TMPyP/PSS) and of TMPyP doped CaCO3 microparticles (CaCO3-TMPyP).

	λ_{max}^{A} (nm)	λ_{max}^{F} (nm)
TMPyP/Water	421.0	Sh; 717.0 (br)
$CaCO_3$-TMPyP	432.0	650.0; 716.0
TMPyP/PSS	430.0	650.0; 713.0

As it can be seen in Table S1, porphyrin adsorption during $CaCO_3$ co-precipitation growth causes a red-shift of 11 nm when compared to water. Major changes are visible in the emission spectra of $CaCO_3$-TMPyP now showing two resolved emission bands at 650 and 716 nm. These spectral features are typical of the formation of a TMPyP-PSS complex (TMPyP/PSS, Table S1) and were previously observed for TMPyP doped polyelectrolyte microcapsules with porphyrin adsorbed in the outer PSS layer. (Reference 22, main manuscript). In the last case, fluorescence lifetimes are reported to be τ = 11.7 ns, which also agrees well with our measured data (τ = 12 ns).

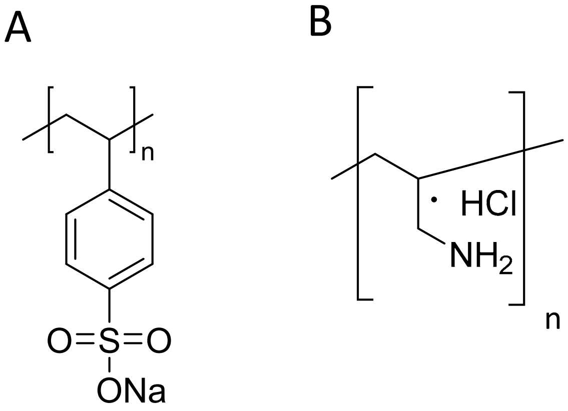

Figure 1. Molecular structures of (**A**) poly(sodium 4-styrenesulfonate)-PSS and (**B**)-poly(allylamine hydrochloride)-PAH.

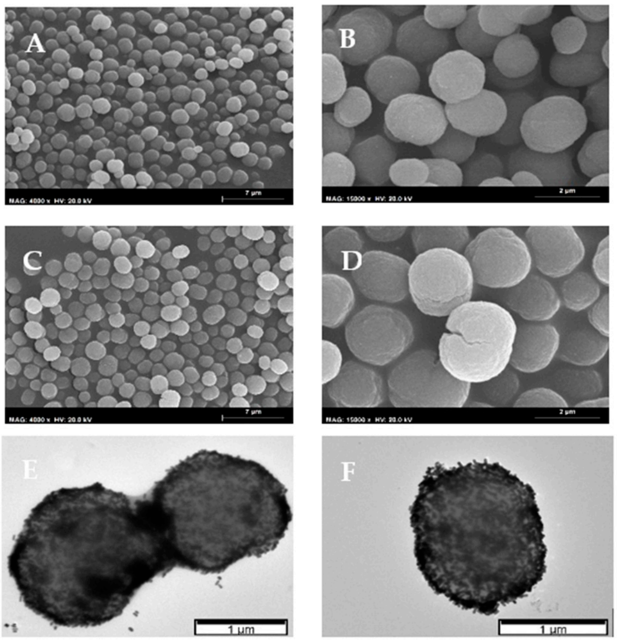

Figure S2. SEM images of (**A**,**B**) non functionalized PECs organized according to ($CaCO_3$)-PSS-PAH (**C**,**D**) one probe polyelectrolyte microcapsules ($CaCO_3$-TMPyP)-PSS-PAH (**E**,**F**) Porphyrin-nanogold PEC's hybrid ($CaCO_3$-TMPyP)-PSS-PAH-(AuNR-PSS).

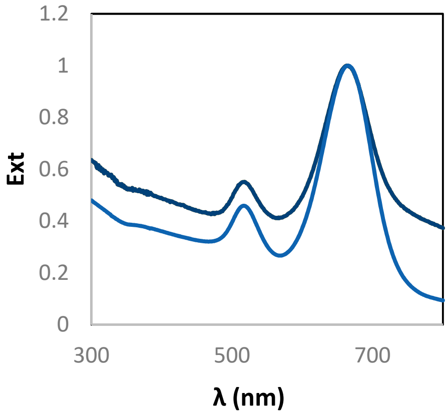

Figure S3. Normalized extinction spectra of (blue) commercially available AuNR and respective PSS wrapped AuNR, AuNR-PSS (black).

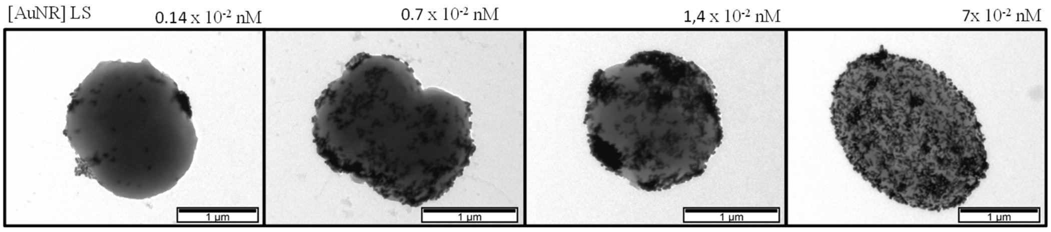

Figure S4. Representative TEM images of TMPyP-nanogold PEC's hybrid obtained by using different concentrations of AuNR-PSS for adsorption.

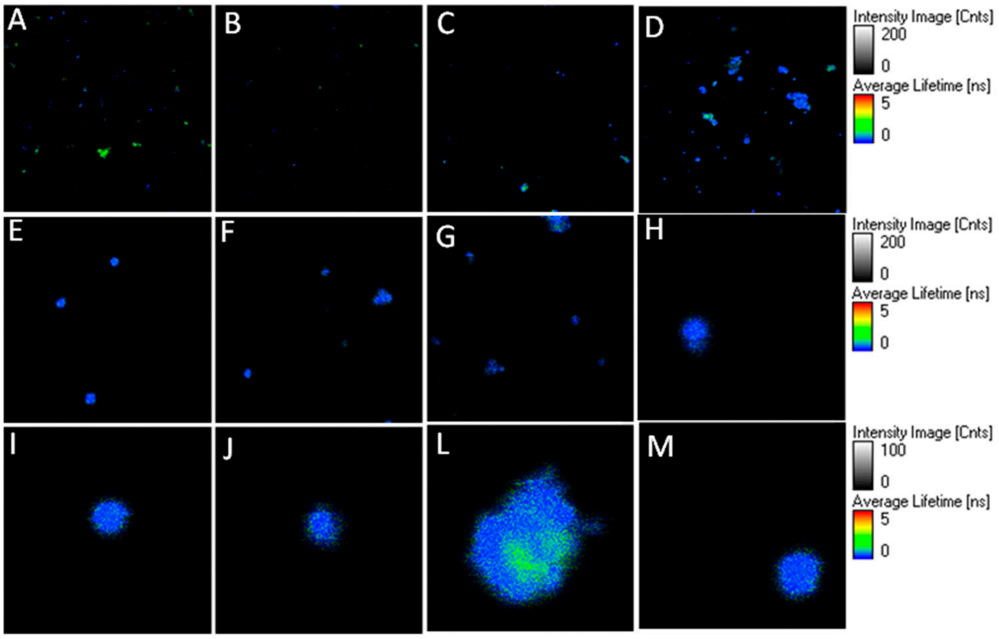

Figure S5. Examples of typical FLIM images measured for ($CaCO_3$-TMPyP)-PSS-PAH-(AuNRPSS): (**A**–**D**) 80 µm ×.80 µm; (**E**–**G**) 20 µm × 20 µm; (**H**–**M**) 5 µm × 5 µm. (λ_{exc} = 639 nm, 0.44 kW/cm^2).

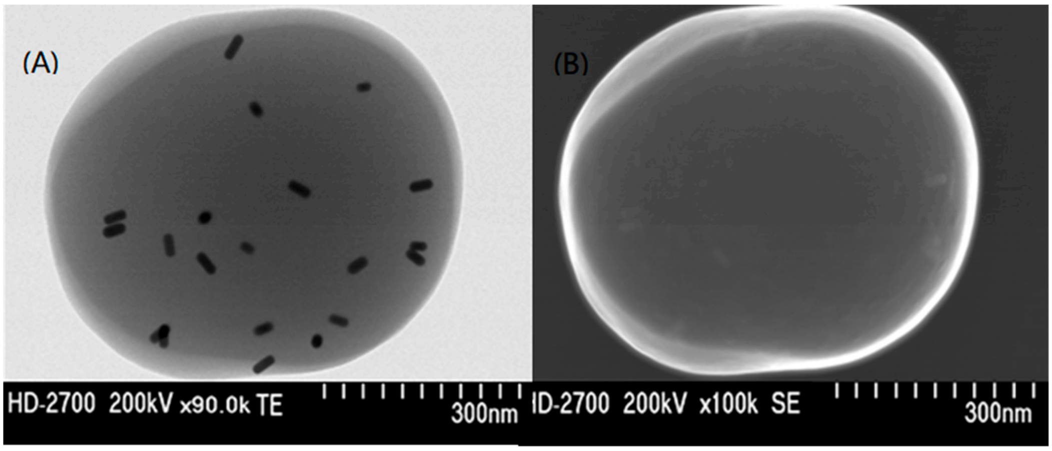

Figure S6. Representative TEM (**A**) and SEM (**B**) images of alternative PEC's hybrid obtained by a co-precipitation approach were $CaCO_3$ growth was promoted in the presence of both TMPyP and gold nanorods, showing a low degree of AuNR adsorption within $CaCO_3$ template and bellow PEC's polyelectrolyte shell.

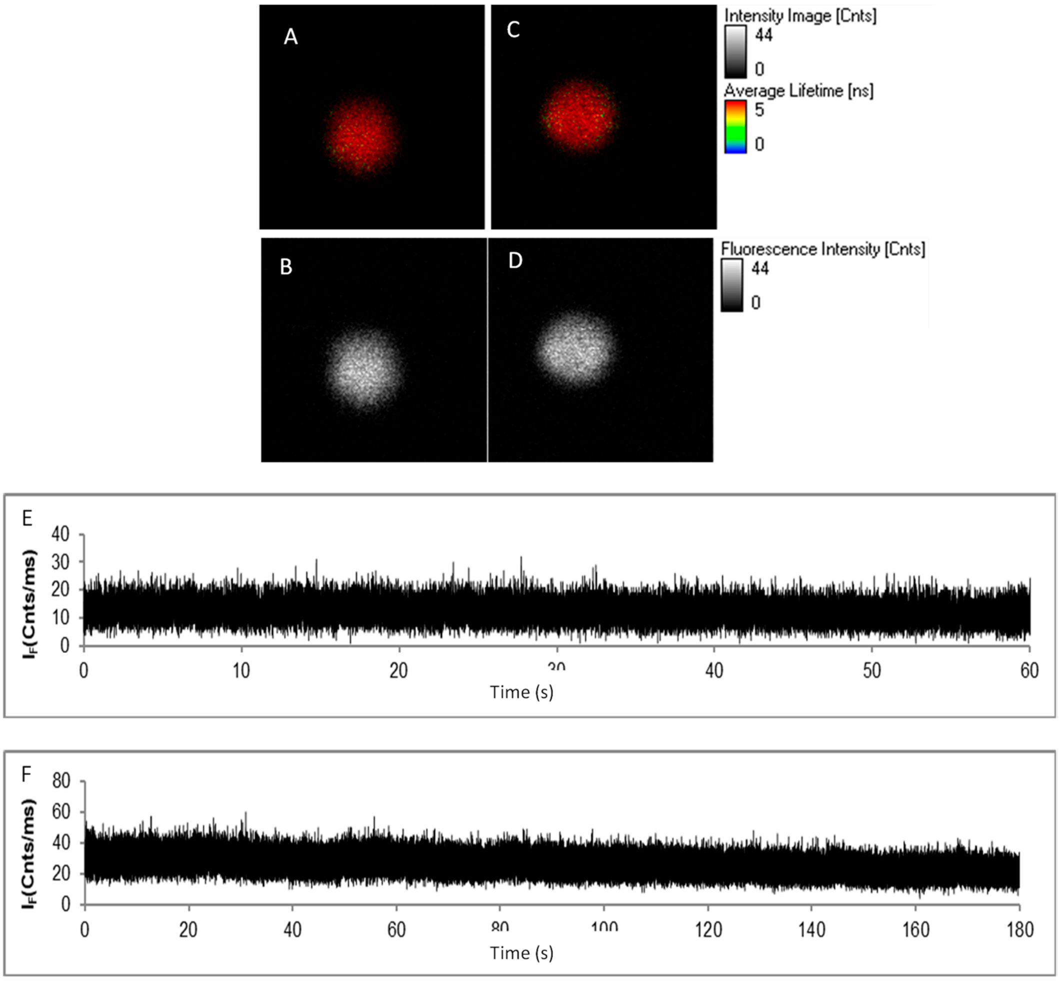

Figure S7. Representative FLIM images obtained for TMPyP doped PECs ($CaCO_3$-TMPyP)-PSS-PAH (**A**,**B**) and for alternative PEC's hybrids obtained by a co-precipitation approach where $CaCO_3$ growth was promoted in the presence of both TMPyP and gold nanorods according to ($CaCO_3$-TMPyP-AuNR)-PSS (**C**,**D**) showing similar porphyrin lifetimes and brightness. (**E**,**F**) Examples of typical intensity time traces found for ($CaCO_3$-TMPyP-AuNR)-PSS.

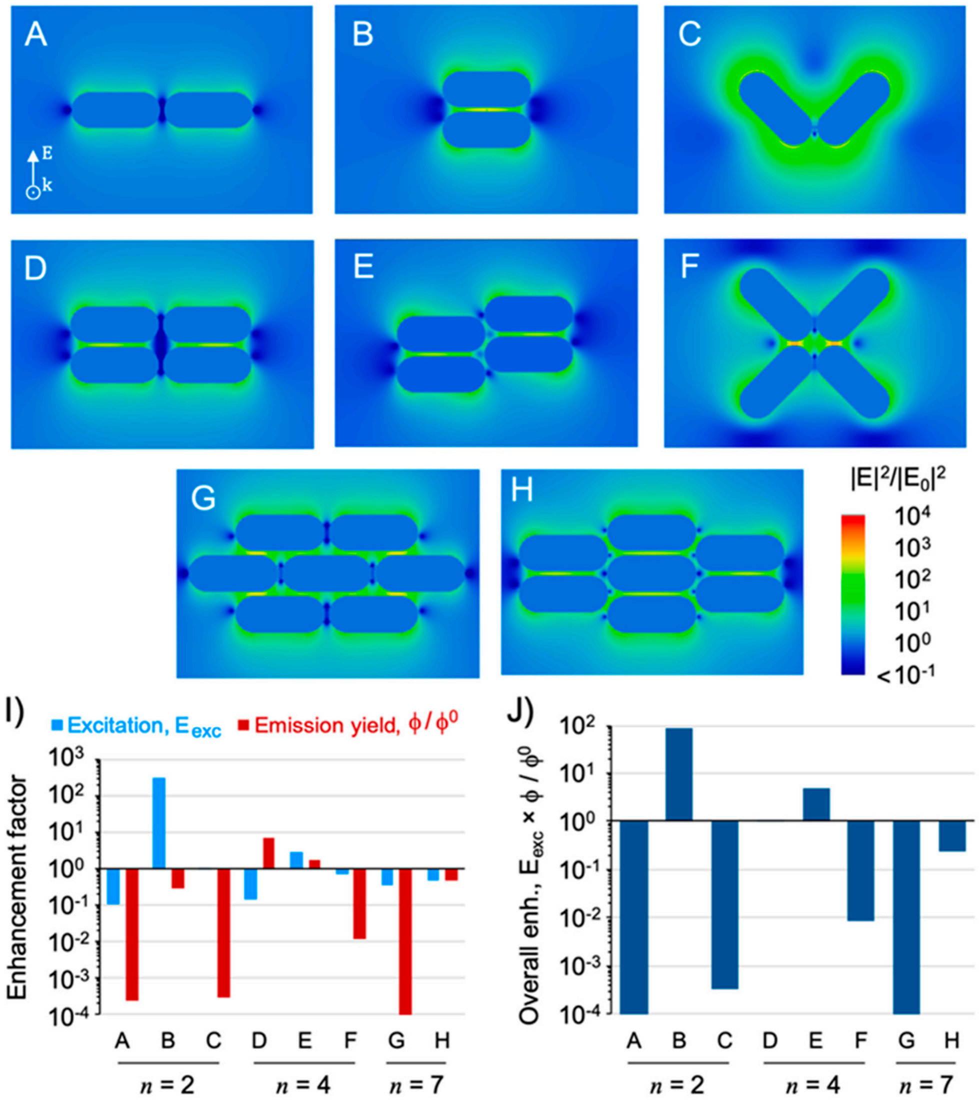

Figure S8. Model simulations of plasmonic hot-spots between AuNRs for particle configurations of (**A**–**C**) two, (**D**–**F**) four and (**G**,**H**) seven nanorods. Herein, the incident field was chosen perpendicular to the longitudinal axis of nanorods (see panel A). (**I**) Calculated enhancement factors for excitation rate, E_{exc}, and for relative emission quantum-yield, ϕ/ϕ^0 (**J**) Overall emission enhancement calculated for a porphyrin-like emitter in selected hot-spots of simulated particle configurations. The emitting dipole was selected in the perpendicular direction to the longitudinal axis of nanorods. In most cases, these simulation results for a perpendicular dipole orientation show a strong quenching effect instead of the large emission enhancements obtained for a parallel dipole orientation, as shown in Figure 5 of the main text.

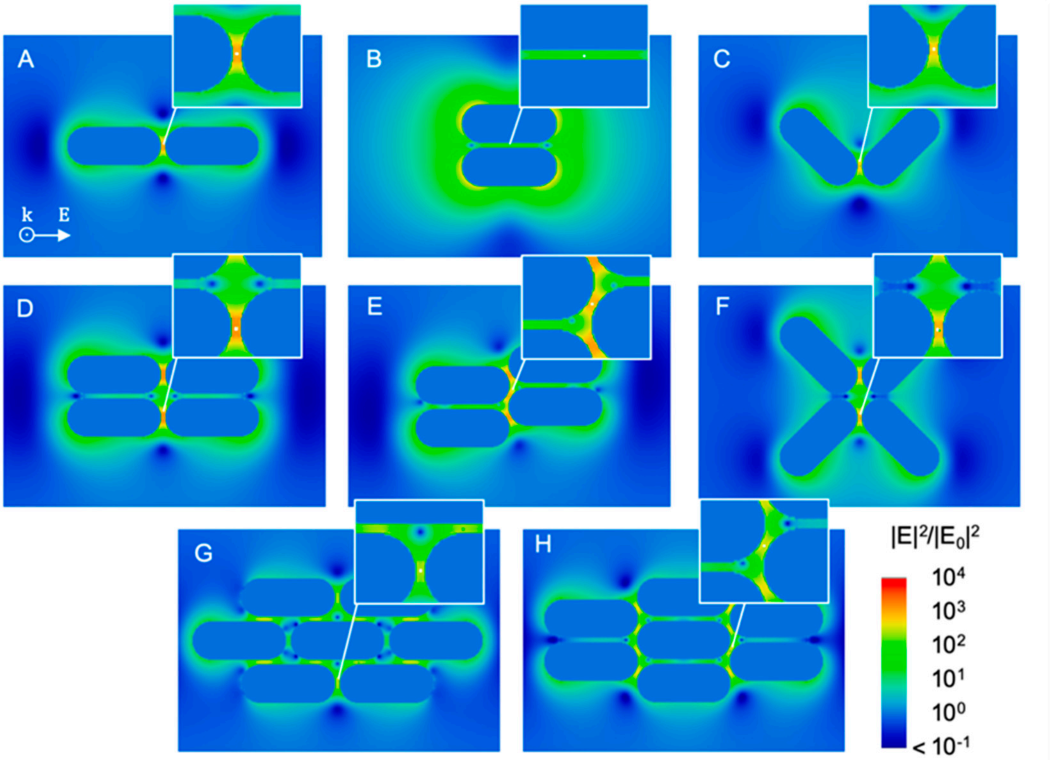

Figure S9. Near-field enhancement maps calculated from model simulations of plasmonic hot-spots between AuNRs for particle configurations of (**A**–**C**) two, (**D**–**F**) four and (**G**,**H**) seven nanorods. The excitation wavelength used in these simulations was 640 nm, in order to match the laser excitation source used in the experiments. The incident field is polarized along the longitudinal axis of nanorods (see panel A). The insets show a zoom-in on the position selected for a point-like dipole emitter (white dot), that simulates the emission of TMPyP porphyrin (see main text for further details).

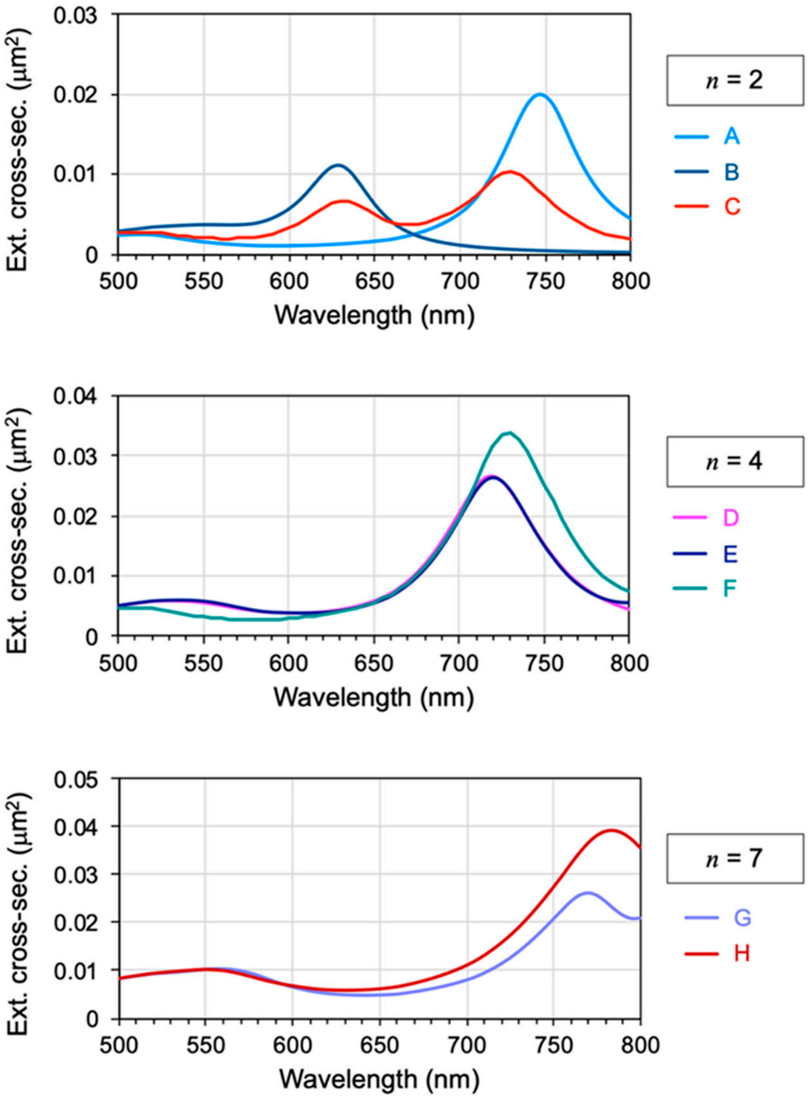

Figure S10. Calculated spectra of particle configurations shown in figure S8 or S9 using the same labelling scheme: A–C (top panel), D–F (middle panel) and G, H (lower panel). The extinction cross-section values are averages for three orthogonal directions. In general, the longitudinal surface plasmon resonance shifts to longer wavelengths with an increasing number of coupled nanorods in end-to-end arrangement.
